# Increasing the transparency of systematic reviews: presenting a generalized registration form

**DOI:** 10.1186/s13643-023-02281-7

**Published:** 2023-09-22

**Authors:** Olmo R. van den Akker, Gjalt-Jorn Ygram Peters, Caitlin J. Bakker, Rickard Carlsson, Nicholas A. Coles, Katherine S. Corker, Gilad Feldman, David Moreau, Thomas Nordström, Jade S. Pickering, Amy Riegelman, Marta K. Topor, Nieky van Veggel, Siu Kit Yeung, Mark Call, David T. Mellor, Nicole Pfeiffer

**Affiliations:** 1 Department of Methodology & Statistics, Tilburg University, Tilburg, The Netherlands; 2 Department of Theory Methodology & Statistics, Open University, Heerlen, The Netherlands; 3 Discovery Technologies Unit, University of Regina, Regina, Canada; 4 Department of Psychology, Linnaeus University, Växjö, Sweden; 5 Center for the Study of Language and Information, Stanford University, Stanford, USA; 6 Department of Psychology, Grand Valley State University, Allendale, USA; 7 Department of Psychology, University of Hong Kong, Hong Kong, Hong Kong; 8 School of Psychology and Center for Brain Research, University of Auckland, Auckland, New Zealand; 9 Department of Psychology, University of York, York, UK; 10 Social Sciences Library, University of Minnesota, Minneapolis, USA; 11 School of Psychology, University of Surrey, Guildford, UK; 12 School of Animal and Human Sciences, Writtle University College, Chelmsford, UK; 13 Center for Open Science, Charlottesville, USA

**Keywords:** Systematic review, Preregistration, Registration, Transparency

## Abstract

This paper presents a generalized registration form for systematic reviews that can be used when currently available forms are not adequate. The form is designed to be applicable across disciplines (i.e., psychology, economics, law, physics, or any other field) and across review types (i.e., scoping review, review of qualitative studies, meta-analysis, or any other type of review). That means that the reviewed records may include research reports as well as archive documents, case law, books, poems, etc. Items were selected and formulated to optimize broad applicability instead of specificity, forgoing some benefits afforded by a tighter focus. This PRISMA 2020 compliant form is a fallback for more specialized forms and can be used if no specialized form or registration platform is available. When accessing this form on the Open Science Framework website, users will therefore first be guided to specialized forms when they exist. In addition to this use case, the form can also serve as a starting point for creating registration forms that cater to specific fields or review types.

## Background

Systematic reviews are systematic in the sense that they involve a systematic process to transparently, reproducibly, and often exhaustively identify and synthesize the literature on a given research topic. Even though objectivity is desirable for systematic reviews, the process is not immune to bias. Systematic reviewers are well aware of this, and many initiatives have been undertaken to identify and prevent biases. In 2011, a registry of systematic reviews (PROSPERO) was created to help researchers prospectively register health-related systematic review protocols [1]. This registry was an important step in making the systematic review process more transparent as it facilitated documentation of the process and justifications for deviations from the protocol. The registry also allowed third parties to check the extent to which completed systematic reviews (as presented in journal articles) are carried out in line with the protocol, making it easier to identify decisions that may have introduced bias (e.g., a change in the criteria for study inclusion or the omission of an analysis without a valid rationale). Finally, PROSPERO allows researchers to check whether similar endeavors are underway prior to engaging in a systematic review, facilitating collaboration and synergy. In all, PROSPERO makes the systematic review process more transparent, and makes it feasible to identify and address biases so that they are less likely to influence the results of systematic reviews.

When registering a systematic review in the PROSPERO registry, researchers are presented with a registration form that they can use to specify their protocol (see Appendix 1 for the PROSPERO registration form). However, this form is optimized for health-related systematic reviews (either in humans or in animals). This serves PROSPERO’s goal well but is necessarily exclusive to other systematic reviews. Specifically, PROSPERO directly excludes all systematic reviews without health outcomes, systematic reviews that are non-interventional, scoping reviews, evidence maps, and qualitative systematic reviews. PROSPERO’s focus on health-related reviews also manifests itself through the items included in the registration form. For example, the form prompts specification of the “disease, condition or healthcare domain being studied”, and the form assumes that some kind of intervention will take place, including mandatory fields where the intervention(s)/exposure(s) and the comparator(s)/control are specified, even though much research does not involve interventions.

To enable researchers to register systematic reviews for which PROSPERO is not suitable, we developed a generalized form for registering systematic reviews that is designed to be applicable across disciplines (i.e., psychology, economics, law, physics, or any other field) and across review types (i.e., scoping review, review of qualitative studies, meta-analysis, or any other type of review). This means that the reviewed records may include research reports as well as archive documents, case law, books, poems, etc**.** Therefore, our selection of items and formulation of each item were optimized for broad applicability instead of specificity. Such generic formulation means some benefits afforded by a tighter focus (e.g., on a given method) may have been forgone. This form, therefore, is well suited as a fallback for more specialized forms and can be used if no specialized form or registration platform is available. When accessing this form on the Open Science Framework website [2] users will therefore first be guided to specialized forms when they exist. If such a specialized form does not exist, we encourage users to reflect on whether this generalized form suits their needs, or whether it would be better to adapt the form into a form that better caters to the user's specific field or review type. As such, this generalized form can also function as a starting point for creating new registration forms. 

To select items for this form, we assessed the items of several reporting guidelines and guides, most notably the Preferred Reporting Items for Systematic reviews and Meta-Analyses (PRISMA) statement. PRISMA was published to help researchers create reproducible reports of their systematic reviews, and with that alleviate biases in the reporting phase of systematic reviews ([3, 4], for an updated version see [5]). Inspired by PRISMA, additional reporting guidelines have been developed for specific disciplines (e.g., ROSES is tailored to systematic reviews in environmental research, and MOOSE is tailored to systematic reviews in epidemiology) and specific types of reviews (e.g., PRISMA-IPD is tailored to systematic reviews of individual participant data, and PRISMA-DTA is tailored to systematic reviews of diagnostic test accuracy). Where the Generalized Systematic Review Registration Form is optimized for accurate and comprehensive a priori documentation of systematic review procedures, reporting guidelines were optimized for application after completion of a systematic review. Because of these different end goals, reporting guidelines like PRISMA lack detail with respect to decisions that are important regarding the planning of a systematic review. In contrast, this form includes several decisions that are important to transparently document before data collection for the systematic review begins. At the same time, some PRISMA items can only be filled out once a systematic review is finished.

Nonetheless, there is also considerable overlap: these reporting guidelines do partly capture the same information as registration forms. Therefore, for each item in this form, we specified the corresponding PRISMA item (PRISMA items P1-P22 and P25-27 were applicable; P16-P23 cover reporting of results and P24 refers to registration forms like this). Researchers planning to use a specific reporting standard to report the results of their review, should enter the information required by that reporting standard in the corresponding (overarching) fields of this form.

The Generalized Systematic Review Registration Form is the result of a collaborative effort of several groups of researchers that independently identified the need for a systematic review registration form that is not restricted to a specific context. These groups initially started to build such forms based on their own research needs but when they learnt about each other’s initiatives through Twitter and academic conferences they decided to combine resources and create this form. These existing resources were the PRISMA statement outlined above, a preregistration template specifically designed for non-interventional research [6], a registration form drafted at the conference of the Society for the Improvement of Psychological Science in 2018, and a registration form drafted for systematic reviews in animal research. We included all items that overlapped between two or more resources in the new form. For the remaining items, we decided collectively through Zoom-meetings and e-mail discussions whether and how to include them. In the final stage, the form was presented to experts in the scientific community to solicit feedback to improve generalizability and usability even more [7]. Based on this feedback, the template was polished into its current state.

## Instructions

To align with general use and open science best practices, when you fill out the form on the Open Science Framework, all items are mandatory. Being as comprehensive as possible makes your registration more useful for readers, funders, yourself, and others, so check carefully whether you did not accidently omit an item. If an item asks about a procedure you do not plan to use or is not applicable, indicate that in the corresponding field (including, ideally, the underlying reason).

You should be transparent about any deviations from the preregistration and provide the rationale for these deviations in your final review. If you already foresee some deviations when filling out the form (e.g., you anticipate that you will not have enough studies in a moderator group), provide a contingency plan for these deviations in the relevant parts of the registration. In addition, we recommend publishing updated registrations, allowing you to document and justify your decisions along the way in the same uniform format.

The aim of this registration form is to be optimally inclusive (i.e., to be usable for registration of any systematic review, regardless of scientific discipline or review type). This inclusivity is also signified by the fact that this form has been used for published papers involving scoping reviews, systematic reviews, narrative reviews, and meta-analyses from areas as diverse as psychology, political science, and biomedicine [8–12]. Moreover, since it was made public on the Open Science Framework (10 April 2023), the form has already been used 68 times as per 2 May 2023, which amounts to more than 20 completed registrations per week. Readers can see how the template is being used in research on the OSF Registry [13]. Given that the form is relatively new and awareness of the form is expected to grow, we expect this average to increase more in later months and years.

Because this aim precludes 1:1 correspondence with the existing reporting guidelines, we want to emphasize that this form is also intended as a basis to develop more specialized forms that do correspond closely to more specific reporting guidelines. Such specialized forms can include, for example, additional fields, added comments, and worked examples. This form is included in the *preregr* R package [14], and the underlying *preregr* form specification [15, 16] can be used to develop adapted versions of this form (e.g., [17]). Note that *preregr* can also be used to produce an R Markdown template containing this form presented in this paper, including the item labels and descriptions using the command “preregr::form_to_rmd_template('genSysRev_v1', file = 'C:/path/to/file.Rmd');”.

The registration form is presented below. The items of the form are denoted by GSRRF, while the corresponding items from the PRISMA checklist are denoted by PRISMA.

## Metadata

This metadata applies only to the registration you are creating, and will not be applied to your OSF project.

**Form version:** v1.00

[GSRRF-1] **Title:**

Prisma: 1.

[GSRRF-2] **Contributors:**

[GSRRF-3] **Subjects:**

[GSRRF-4] **Tasks and roles:**

Describe the expected tasks and roles of each author/contributor, for example using the Contributor Roles Taxonomy (CRediT).

## Review methods

In this section, you register the general type, background and goals of your review.

[GSRRF-5] **Type of review:**

This can be, for example, a meta-analysis, evidence map, or a qualitative review. Also indicate whether you used any guidelines, tools or checklists to prepare your protocol, and if so, which ones. For more information, see: Tricco, A. C., Tetzlaff, J., Moher, D. (2011). The art and science of knowledge synthesis. https://doi.org/frdpd2. PRISMA: 1.

[GSRRF-6] **Review stages:**

Indicate the stages in which you will conduct this review. Common stages are, in this order, the sections of this form: Search, Screening, Extraction, Synthesis. Sometimes other stages are distinguished, such as Preparation, Critical Appraisal, and Reporting. Additionally, it can be beneficial to include pilot stages for screening and extraction, while mentioning any updates to the preregistration. The stages could then look like: Preparation, Search, Pilot Screening (100 hits), Prereg Update, Screening, Pilot Extraction (10 sources), Prereg update, Extraction, Synthesis.

[GSRRF-7] **Current review stage:**

Indicate in which stage from the list you specified in the “Review stages” item you are at this moment (i.e., when you freeze this registration). Note that in many contexts, only registrations in earlier stages count as preregistrations. For example, you can use a table to indicate whether you started and/or finished with a certain stage as is customary for PROSPERO registrations. In addition, if this is not the first preregistration (but a second or third update, e.g., after pilot screening or pilot extraction), you can make that explicit here.

[GSRRF-8] **Start date:**

Indicate the planned start date, or if you already started, the actual start date.

[GSRRF-9] **End date:**

Indicate the planned end date, or if you already completed the review, the actual end date. You can use resources such as PredicTER.org to estimate how long a review will take to complete.

[GSRRF-10] **Background:**

Introduce the topic of your review, its aims, and/or provide a short summary of known literature and what your review adds to this literature. You can describe why the review is needed, as well as which reviews already exist on this or related topics. PRISMA: 2 and 3.

[GSRRF-11] **Primary research question(s):**

List the specific questions this review is meant to answer (i.e., the questions that ultimately informed the decisions made when designing the search strategy, and screening, extraction, and synthesis plans). You may find it helpful to refer to frameworks such as PICOS where appropriate to pinpoint your research questions. Note that all analyses pertaining to primary research questions should normally be reported in the final report. PRISMA: 4.

[GSRRF-12] **Secondary research question(s):**

List additional research questions that you will examine, but that took less central roles in informing the review’s design. Note that all analyses pertaining to secondary research questions should normally be reported in the final report. PRISMA: 4.

[GSRRF-13] **Expectations / hypotheses:**

Describe any hypotheses (common for quantitative approaches) and/or expectations you have. These can pertain to your research questions, the types of sources you will find, social and political contexts, and contextual information that you know may color your interpretations and decisions (common for qualitative approaches). PRISMA: 3

[GSRRF-14] **Dependent variable(s) / outcome(s) / main variables:**

List the dependent / outcome / main variables you are interested in. If this review concerns one or more associations, list the outcome variable(s) or dependent variables. If this review does not concern one or more associations (e.g., in reviews of single variables such as prevalences, or descriptive reviews), list the main variables of interest here. PRISMA: 10a.

[GSRRF-15] **Independent variable(s) / intervention(s) / treatment(s):**

If this review’s research question(s) concerns one or more associations or effects, list the variable(s) that theoretically cause them or are assumed to otherwise explain the dependent variable(s) / outcome(s). If this is a manipulation, treatment, or intervention, make sure to describe it in full: that means also describing all groups, including any control group(s) or comparator(s). PRISMA: 10b.

[GSRRF-16] **Additional variable(s) / covariate(s):**

Here, list any additional variables you are interested in that were not included in the two lists above, such as covariates, moderators, or mediators. PRISMA: 10b.

[GSRRF-17] **Software:**

List the software you will use for the review, for instance to store and screen search results, extract data, keep track of decisions, and to synthesize the results. Include version numbers and the operating systems, if applicable. PRISMA: 13d.

[GSRRF-18] **Funding:**

List the funding sources for everybody that is involved in this review at this stage. If the work is unfunded, please state this as such. PRISMA: 25.

[GSRRF-19] **Conflicts of interest:**

List any potential conflicts of interest (e.g., if there is a potential outcome of this review that can in any way have negative or positive effects for anybody involved in this review in terms of funding, prestige, or opportunities). If there are no conflicts of interest, please state this as such. PRISMA: 26.

[GSRRF-20] **Overlapping authorships:**

Declare whether you expect that anyone involved in this review is a co-author of one of the studies that will likely be included in the review (based on your search strategy) and, if so, how you will address potential bias (i.e., that reviewer is not involved in screening, data extraction, quality assessment, or synthesis of that study). If you are confident that this does not represent a conflict of interest, explain why you think so. PRISMA: 26.

## Search strategy

In this section, you register your search strategy: the procedures you designed to obtain all (potentially) relevant sources to review (e.g., articles, books, preprints, reports, case law, policy papers, archived documents).

[GSRRF-21] **Databases:**

List the databases you will search (e.g., ArXiv, PubMed, Scopus, Web of Science, PsycINFO, AGRIS, BioOne, PubChem). Note that these are different from interfaces (see below and here). PRISMA: 6.

[GSRRF-22] **Interfaces:**

For each database, list the interface you used to search that database (e.g., Ovid or EBSCO). Some databases are provided by the same organisation, in which case the interface can have the same name (e.g., PubMed, ArXiv). For more information about the distinction, see here. PRISMA: 6.

[GSRRF-23] **Grey literature:**

List your strategies for locating grey literature (i.e., sources not indexed in the databases you search) such as pre-prints (e.g., disciplinary repositories such as ArXiv or PsyArXiv or university repositories using for example, Dspace), dissertations and theses, conference proceedings and abstracts, government/industry reports etc. PRISMA: 6.

[GSRRF-24] **Inclusion and exclusion criteria:**

List the specific inclusion and exclusion criteria that you used to inform your search strategy. Also list the framework(s) you used to establish your exclusion and inclusion criteria and use them to develop your search query, if any. Examples of the latter are PICO (Population, Intervention, Comparison, Outcome) and SPIDER (Sample, Phenomenon of Interest, Design, Evaluation, Research type), but many more exist (see here for an overview based on the medical and health sciences). PRISMA: 5 and 13a.

[GSRRF-25] **Query strings:**

For each database/interface combination, list the query you will input (note that the available fields and operators can differ by database and by interface). The query string can be based on, for example, your inclusion criteria, the entities you want to extract (see “extraction”) and design requirements (e.g., qualitative studies, RCTs, or prevalence studies). PRISMA: 7.

[GSRRF-26] **Search validation procedure:**

Explain whether you plan to employ a search validation procedure, and if so, describe the procedure. Templates are available here. PRISMA: 7.

[GSRRF-27] **Other search strategies:**

List any additional search strategies you aim to employ, such as using the ascendancy approach (look through other sources cited in your included sources), the descendancy approach (look through the sources that cite your included sources using systems such as Crossref), or using other systems such as CoCites. PRISMA: 7.

[GSRRF-28] **Procedures to contact authors:**

Describe your procedures for contacting authors. Will you contact authors? When? How will you follow-up on your first contact? Do you plan to share meta-data about those communications, and if so, how do you ask authors’ permission for that? Note that templates are available at https://osf.io/q8stz/. PRISMA: 7.

[GSRRF-29] **Results of contacting authors:**

Describe whether you plan to report the outcomes of contacting the authors (e.g., how many authors responded, how many authors sent data), and if so, how. PRISMA: 16a.

[GSRRF-30] **Search expiration and repetition:**

Depending on how quickly the literature in an area expands, searches can have limited expiration dates; and for living reviews, repetition is planned regardless of ideas about expiration. Will you repeat your search (for example, in the case of a living review), and if so, how many months or years after your first search? PRISMA: 7.

[GSRRF-31] **Search strategy justification:**

Search strategies are often compromises, balancing pragmatic considerations with scientific rigour. Here, describe the justifications for your decisions about the databases, interfaces, grey literature strategies, query strings, author contact procedures, and search expiration date. PRISMA: 7.

[GSRRF-32] **Miscellaneous search strategy details:**

Here, you can describe any details that are not captured in the other fields in this section. PRISMA: 7.

## Screening

In this section, you register your screening procedure: the procedure you designed to eliminate all irrelevant sources from the results of the search strategy (and retain the relevant sources).

[GSRRF-33] **Screening stages:**

Describe the stages you will use for screening. For example, if you expect many hits, you may want to first screen based on titles only, in a second round also include abstracts and keywords, and in a third round screen based on full texts. Also indicate for each round whether the screening is done by a computer (e.g., AI), a human, or a computer supervised by a human. Don’t forget to describe the deduplication procedure, if you implement it. PRISMA: 8.

[GSRRF-34] **Screened fields / blinding:**

Describe which bibliographic fields (e.g., title, abstract, authors) are visible during the screening, and which fields are blinded. For example, journal names, authors, and publication years can be hidden from screeners in an effort to minimize bias. PRISMA: 8.

[GSRRF-35] **Used exclusion criteria:**

List the specific exclusion criteria that you apply in your screening to eliminate sources from the set of sources identified in your search. Note that inclusion criteria are typically used to inform the search strategy; during screening, as soon as an exclusion criterion is met, an entry is excluded, and so, inclusion criteria are reformulated into exclusion criteria where applicable. PRISMA: 8.

[GSRRF-36] **Screener instructions:**

List or upload the instructions provided to the screener(s). PRISMA: 8.

[GSRRF-37] **Screening reliability:**

For each screening round, list the number of screeners and the procedure used to ensure independent screening.This can also mean that you declare that you only use one screener, use multiple screeners that work together, or that you will not implement procedures to ensure that the screening is conducted independently. Also explain the test you will use, if any, to assess screener agreement. PRISMA: 8.

[GSRRF-38] **Screening reconciliation procedure:**

If you use more than one screener, describe the procedure to deal with divergent screener decisions for each screener round (e.g., through discussion or input from an additional screener). PRISMA: 8.

[GSRRF-39] **Sampling and sample size:**

Describe whether you plan to use all sources included through the screening procedure, or whether you plan to sample from these sources (note that in most cases, all studies identified at this stage are kept). In case of the latter, describe the procedure you plan to use, the sample size analyses you conducted or will conduct, and the resulting required sample size if that is already available. If you plan to refrain from drawing conclusions, or draw more nuanced conclusions, describe that here as well. Finally, describe what you will do if a minimum required sample size or power is not reached (for your main analysis and any supplementary analyses). PRISMA: 8.

[GSRRF-40] **Screening procedure justification:**

Screening procedures are often compromises, balancing pragmatic considerations with scientific rigour. Here, describe the justifications for your decisions about the screening rounds, blinding, in/exclusion criteria, assurance, and reconciliation procedures. PRISMA: 8.

[GSRRF-41] **Data management and sharing:**

Describe whether and how you plan to share the sources you obtained from the searches in the databases (see Search Strategy) and the decisions each screener made in each screening round. List both the file format (e.g., BibTeX, RIS, CSV, XLSX), the repository, and any potential embargos or conditions for access. PRISMA: 27.

[GSRRF-42] **Miscellaneous screening details:**

Here, you can describe any details that are not captured in the other fields in this section. PRISMA: 8.

## Extraction

In this section, you register your plans for data extraction: the procedures you designed to extract the data you are interested in from the included sources. Examples of such data are text fragments, effect sizes, study design characteristics, year of publication, characteristics of measurement instruments, final verdicts and associated penalties in a legal system, company turnovers, sample sizes, or prevalences.

[GSRRF-43] **Entities to extract:**

List all entities that will be extracted from each included source. Entities can be, for example, 1) variables such as values of independent and dependent variables, and potential moderators (e.g., means, standard deviations); 2) estimations of associations between variables or effect sizes (e.g., Pearson’s r or Cohen’s d); 3) qualitative data fragments (e.g., interview material or synthesized themes); 4) descriptions of the used methods such as the included studies’ designs, sample sizes, sample characteristics, time between data collection sessions, and blinding procedures; 5) metadata such as authors, institutions, and year of publication; 6) and (other) risk of bias indicators. PRISMA: 10a, 10b, and 12.

[GSRRF-44] **Extraction stages:**

Describe the stages you will use for extraction. Examples of stages are: a training stage, a reliability verification stage, and a final extraction stage; or first extracting primary data and in a second stage risk of bias information; or two extractors working sequentially or in parallel. Also indicate for each stage whether the extraction is done by a computer (e.g., AI), a human, or a computer supervised by a human. PRISMA: 9.

[GSRRF-45] **Extractor instructions:**

List or upload the instructions provided to the extractors (i.e., those performing the data extraction). PRISMA: 9.

[GSRRF-46] **Extractor blinding:**

If blinding is used, describe the procedure used to blind extractors from the research questions, hypotheses, and/or specific roles of each entity to extract in this review. For example, extractors can be research assistants who are not informed of the study’s background or research questions, but who are trained to extract entities using the coding instructions you developed for each entity; or entity extraction can be crowdsourced to citizen scientists. PRISMA: 9.

[GSRRF-47] **Extraction reliability:**

For each extraction round, list the number of extractors and the procedure used to ensure independent extraction (this can also mean that you declare that you use one extractor, or will not implement procedures to ensure that the extractions are conducted independently). Also explain the test you will use, if any, to assess extractor agreement. PRISMA: 9.

[GSRRF-48] **Extraction reconciliation procedure:**

For each extraction round, describe the procedure to deal with divergent extraction decisions (if applicable, i.e., if you use more than one extractor). PRISMA: 9.

[GSRRF-49] **Extraction procedure justification:**

Extraction procedures are often compromises, balancing pragmatic considerations with scientific rigour. Here, describe the justifications for your decisions about the justification of each entity that will be extracted, the extraction rounds, reliability assurance, and reconciliation procedures. PRISMA: 9.

[GSRRF-50] **Data management and sharing:**

Describe whether and how you will share the files with the extracted entities (as specified in the corresponding field above; i.e., everything extracted from every source, including metadata, method characteristics, variables, associations, etc). List both the file format (e.g., CSV, XLSX, Rdata), the repository, and any potential embargos or conditions for access. Describe efforts made to share FAIR, 5-star open data, if any such efforts will be made. PRISMA: 27.

[GSRRF-51] **Miscellaneous extraction details:**

Here, you can describe any details that are not captured in the other fields in this section. PRISMA: 9.

**Synthesis and Quality Assessment**

In this section, you register the procedure for the review’s synthesis: the procedure you designed to use the data that was extracted from each source to answer your research question(s). This often includes transforming the raw extracted data, verifying validity, applying predefined inference criteria, interpreting results, and presenting results. Additionally, you register procedures you designed to assess bias in individual sources and the synthesis itself.

[GSRRF-52] **Planned data transformations:**

Describe your plans for transforming the raw extracted data. This may include converting effect sizes to other metrics (e.g., convert all metrics to Pearson correlation coefficients); recoding or (re)categorizing extracted qualitative data fragments (e.g., coding extracted music genres within an existing taxonomy); and aggregating extracted data prior to the main synthesis procedures (e.g., compute the mean of a variable over all samples in one source). Applying these transformations to the raw extracted entities from the Extraction section should yield data that corresponds to the variables of interest listed in the Review Methods section. PRISMA: 13b.

[GSRRF-53] **Missing data:**

Describe how you will deal with missing data (i.e., cases where it is not possible to extract one or more entities from the source material, and your efforts to obtain the missing information, for example by contacting the authors, are not fruitful). PRISMA: 10b.

[GSRRF-54] **Data validation:**

Describe your process of ensuring that the data are correct and useful (e.g., identifying outliers, identifying retractions, or triangulating with other sources). Also describe your criteria for assessing data validity and how you will deal with data violating those criteria. PRISMA: 10b.

[GSRRF-55] **Quality assessment:**

Describe the analyses you plan to do to assess and weigh the quality of the included sources with respect to your research question(s). Examples of tools to use for quality evaluation are Cochrane’s Risk of Bias 2 tool, GRADE, and GRADE-CERQual. PRISMA: 11.

[GSRRF-56] **Synthesis plan:**

Describe the specific procedure you will apply to arrive at an answer to the research question(s). For example, in meta-analyses this is the full analysis plan, including any planned subgroup analyses and moderator analyses, the (multilevel) model specification, and preferably the analysis code. For a qualitative review, it is the procedure you plan to use to collate your results into a coherent picture. If you distinguish synthesis tiers (e.g., primary and secondary analysis, or confirmatory and exploratory analyses), list them and indicate which procedures you plan to use for each. Also specify what you will do if parts of the plan can’t be properly executed. PRISMA: 13c, 13d, and 13e.

[GSRRF-57] **Criteria for conclusions / inference criteria:**

If you plan to draw your conclusions based on pre-specified criteria (e.g., a minimal effect size of interest, a significance level, or a saturation point), list these here. PRISMA: 20b.

[GSRRF-58] **Synthesist blinding:**

Describe the procedure, if any, used to blind synthesists (i.e., the persons synthesizing the extracted data to arrive at answers to your research question(s)) from the research questions, hypotheses, and/or specific roles of each extracted entity/variable in this review. For example, for meta-analyses, an analyst external to the main research team can be engaged to perform the analyses without knowing the study’s hypotheses. For qualitative reviews, for the synthesis, other researchers can be involved who are unaware of and are not informed about the research process and expectations. PRISMA: 13d.

[GSRRF-59] **Synthesis reliability:**

List the number of synthesists and the procedure used to ensure independent synthesis (this can also mean that you declare that you use one synthesist, or will not implement procedures to ensure that the syntheses are conducted independently). PRISMA: 13d.

[GSRRF-60] **Synthesis reconciliation procedure:**

Describe the procedure to deal with divergent synthesis decisions (if relevant). PRISMA: 13d.

[GSRRF-61] **Publication bias analyses:**

Describe the analyses you plan to do to assess publication bias (if any). For an overview of commonly used publication bias correction methods, see Table 1 in Van Aert, Wicherts, & Van Assen (2019) PRISMA: 14.

[GSRRF-62] **Sensitivity analyses / robustness checks:**

Describe the sensitivity analyses or robustness checks you plan to conduct (if any). PRISMA: 13f and 15.

[GSRRF-63] **Synthesis procedure justification:**

Extraction procedures are sometimes compromises, balancing pragmatic considerations with scientific rigour. Here, describe the justifications for your decisions about your planned transformations (e.g., if based on assumptions, how do you know those are feasible), your data integrity and missing data checks and corrections, your synthesis plan, the criteria you chose to drive your conclusions/inferences (if any), and your procedures for blinding, and reliability assurance/reconciliation if you use multiple synthesists. PRISMA: 13d.

[GSRRF-64] **Synthesis data management and sharing:**

Describe whether and how you will share the files with the analysis scripts, notes, and outputs. List both the file format (e.g., R scripts, Rmarkdown files, plain text files, Open Document files), the repository, and any potential embargos or conditions for access. See here for a generic example of an analysis script. PRISMA: 27.

[GSRRF-65] **Miscellaneous synthesis details:**

Here, you can describe any details that are not captured in the other fields in this section. PRISMA: 13d.

## Data Availability

Not applicable.

## Appendix 1: The PROSPERO registration items

### Review title

This is a mandatory field

Give the working title of the review, for example the one used for obtaining funding. Ideally the title should state succinctly the interventions or exposures being reviewed and the associated health or social problems. Where appropriate, the title should use the PI(E)COS structure to contain information on the Participants, Intervention (or Exposure) and Comparison groups, the Outcomes to be measured and Study designs to be included.

Acronyms may be included in titles, but should not be used alone without expansion unless they are regarded as more usual than the expansion (e.g. HIV).

The title in this field must be in English. If the original title is in a different language the English version must be entered here, with the non-English version entered into the field labelled “Original Language Title”.

If the final title of the review differs, this can be displayed in the Publication of Final Report Field.

Example: Systematic review and meta-analysis of recurrence and survival following pre- versus post-operative radiation in localized, resectable soft-tissue sarcoma.

### Original language title

For reviews in languages other than English, this field should be used to enter the title in the language of the review. This will be displayed together with the English language title.

Example: Revisión sistemática y meta-análisis de la recurrencia y la supervivencia tras la fase de radiación en comparación con post-operatorio en el sarcoma localizados resecables de tejido blando.

### Anticipated or actual start date

This is a mandatory field.

Give the date when the systematic review commenced, or is expected to commence.

For the purposes of PROSPERO, the date of commencement for the systematic review can be defined as any point after completion of a protocol but before formal screening of the identified studies against the eligibility criteria begins.

A protocol can be deemed complete when it is approved by a funder or the person commissioning the review; when peer review is complete; when the protocol is published or when the authors decide that it is complete and they do not anticipate any major revisions to the design of the systematic review.

This field may be edited at any time. All edits to published records will appear in the record audit trail. A brief explanation of the reason for changes should be given in the Revision Notes facility.

Example: 01 June 2011.

### Anticipated completion date

This is a mandatory field.

Give the date by which the review is expected to be completed. In the absence of an agreed contractual date, a realistic anticipated date for completion should be set. It can be modified should the schedule change. When this date is reached, the named contact will receive an automated email to ask them to provide an update on progress.

This field may be edited at any time. All edits will appear in the record audit trail. A brief explanation of the reason for changes should be given in the Revision Notes facility.

Example: 01 June 2011.

### Stage of review at time of this submission.

This is a mandatory field.

Indicate the stage of progress of the review by ticking the relevant Started and Completed boxes. Additional information may be added in the free text box provided.

Please note: Reviews that have progressed beyond the point of completing data extraction at the time of initial registration are not eligible for inclusion in PROSPERO. Should evidence of incorrect status and/or completion date being supplied at the time of submission come to light, the content of the PROSPERO record will be removed leaving only the title and named contact details and a statement that inaccuracies in the stage of the review date had been identified.

This field should be updated when any amendments are made to a published record and on completion and publication of the review.

Example: Preliminary searches ticked as completed, pilot of the study selection process ticked as started.

### Named contact

This is a mandatory field

The named contact acts as the guarantor for the accuracy of the information presented in the register record. This should be the lead reviewer or a representative of the review team. This person is also responsible for submitting details of any amendments while the review is ongoing and publication details after the review is completed. The named contact is the person to whom users of PROSPERO would send questions or comments.

This field is automatically populated from the named contact’s signing in details. The named contact’s name will be displayed in the public record.

Example: Dr Joseph Bloggs.

N.B. To change the named contact for a published record, send details of the existing and new contact to crd-register@york.ac.uk.

### Named contact email

This is a mandatory field

Give the electronic mail address of the named contact. This may be a generic email address to which the named contact has access.

This field is automatically populated from the named contact’s joining details, but can be changed if required. The email address supplied here will be displayed in the public record.

Examples: joseph.bloggs@city.ac.uk or research.secretary@city.ac.uk.

### Named contact address

PLEASE NOTE this information will be published in the PROSPERO record so please do not enter private information, i.e. personal home address.

Give the full postal address for the named contact. (N.B. This field is automatically populated from the named contact’s joining details.)

This address will be displayed in the public record. If you do not wish it to appear in the public record delete the content of this field.

Example: Alcuin B Block,University of York, York, YO10 5DD, UK.

### Named contact phone number

Give the telephone number for the named contact, including international dialling code.

(N.B. This field is automatically populated from the named contact’s joining details.)

This number will be displayed in the public record. If you do not wish it to appear in the public record delete the content of this field.

Example: + 44 (0)10,904 321,040.

### Organisational affiliation of the review

This is a mandatory field

Full title of the organisational affiliations for this review and website address if available. This field may be completed as ‘None’ if the review is not affiliated to any organisation.

Example: Andalusian Agency for Health Technology Assessment (AETSA).

### Review team members and their organisational affiliations

This is a mandatory field

Give the personal details and the organisational affiliations of each member of the review team. Affiliation refers to groups or organisations to which review team members belong. **NOTE: email and country are now mandatory fields for each person.**. Affiliation refers to groups or organisations to which review team members belong.

Review team members will be listed ‘manuscript’ style in the order entered in this list. The named contact will be automatically added to this field, but can be deleted if not a member of the review team. To place the named contact somewhere other than first in order, delete the automatic entry and enter members’ details in the required order.

Membership of the review team and details of affiliations can be updated at any time.

All edits will appear in the record audit trail.

Example: Mr Joseph Bloggs, Centre for Reviews and Dissemination, University of York, UK. Dr Jane Smith, Department of Health Sciences, University of York, UK. Prof. Steven Jones, Centre for Health Statistics, Medical Research Centre, Canada.

### Funding sources/sponsors

This is a mandatory field

Give details of the individuals, organizations, groups or other legal entities who take responsibility for initiating, managing, sponsoring and/or financing the review. Include any unique identification numbers assigned to the review by the individuals or bodies listed.

Examples: NIHR HTA Programme (Project ref 09/13/02). The Terry Fox New Frontiers Program in Cancer (Ref 201006TFL). Funding provided by Amgen, Merck, Roche, and Sanofi-aventis.

### Conflicts of interest

This is a mandatory field

List any conditions that could lead to actual or perceived undue influence on judgements concerning the main topic investigated in the review. The conflicts of interest listed should cover the review team as a whole, as well as individuals in the team.

Conflicts of interest arise when a team member or the team as a whole (e.g. because of the team’s institution) has financial or personal relationships that may inappropriately influence (bias) their actions (such relationships are also known as dual commitments, competing interests, or competing loyalties).These relationships vary from being negligible to having great potential for influencing judgement. Not all relationships represent true conflict of interest.

On the other hand, the potential for conflict of interest can exist regardless of whether a person believes that the relationship affects his or her scientific judgement. Financial relationships (such as employment, consultancies, stock ownership, honoraria, and paid expert testimony) are the most easily identifiable conflicts of interest and the most likely to undermine the credibility of the review.

However, conflicts can occur for other reasons, such as personal relationships, academic competition, and intellectual passion. For the purposes of disclosure, the term “competing interest” should be considered synonymous with conflict of interest.^1^

Example: The lead reviewer (JB) has given talks on this topic at workshops, seminars, and conferences for which travel and accommodation has been paid for by the organisers. The other authors declare that they have no known conflicts of interest.

### Collaborators

Give the name and affiliation of any individuals or organisations who are working on the review but who are not listed as review team members. **NOTE: email and country are now mandatory fields for each person.**

Example: Dr Eric Porter, Oncologist, University Hospital, Brighton, UK. Clinical advisor.

### Review question

This is a mandatory field

State the question(s) to be addressed by the review, clearly and precisely. Review questions may be specific or broad. It may be appropriate to break very broad questions down into a series of related more specific questions. Questions may be framed or refined using PI(E)COS where relevant.

Example: How does pre-operative chemotherapy impact on survival of early stage non-small cell lung cancer compared to surgery alone?

### Searches

This is a mandatory field

State the sources that will be searched. Give the search dates, and any restrictions (e.g. language or publication period). Do NOT enter the full search strategy (it may be provided as a link or attachment.)

The search strategy reported in systematic review protocols should:

- Name all sources that will be used to identify studies for the systematic review.

Sources include (but are not limited to) bibliographic databases, reference lists of eligible studies and review articles, key journals, conference proceedings, trials registers, Internet resources and contact with study investigators, experts and manufacturers.

Systematic reviews typically use more than one database. Examples of electronic bibliographic databases include MEDLINE, EMBASE, PsycINFO. Other database sources include The Cochrane Library, Health Technology Assessment Database, and Web of Science.

- Search dates (from and to)
- Restrictions on the search including language and publication period
- Whether searches will be re-run prior to the final analysis

It is considered good practice for searches to be re-run just before the final analyses and any further studies identified, retrieved for inclusion.

- Whether unpublished studies will be sought

### URL to search strategy

Give a link to the search strategy or an example of a search strategy for a specific database if available (including the keywords that will be used in the search strategies).

Alternatively, an electronic file could be supplied which will be linked to from the Register record. This will be made publicly available from the published record immediately, or it can be held in confidence until the review has been completed, at which time it will be made publicly available.

Example: http://www.biomedcentral.com/1756-0500/3/250

### Condition or domain being studied

This is a mandatory field

Give a short description of the disease, condition or healthcare domain being studied. This could include health and wellbeing outcomes.

Examples: Type 2 diabetes. Physical activity in children.

### Participants/population

This is a mandatory field

Give summary criteria for the participants or populations being studied by the review. The preferred format includes details of both inclusion and exclusion criteria.

Example:

Inclusion: Adults with schizophrenia (as diagnosed using any recognised diagnostic criteria).

Exclusion: Adolescents (under 18 years of age) and elderly people (over 70).

### Intervention(s), exposure(s)

This is a mandatory field

Give full and clear descriptions or definitions of the nature of the interventions or the exposures to be reviewed. This is particularly important for reviews of complex interventions (interventions involving the interaction of several elements). If appropriate, an operational definition describing the content and delivery of the intervention should be given.

Ideally, an intervention should be reported in enough detail that others could reproduce it or assess its applicability to their own setting. The preferred format includes details of both inclusion and exclusion criteria.

For reviews of qualitative studies give details of the focus of the review.

Example: Population-level tobacco control interventions are defined as those applied to populations, groups, areas, jurisdictions or institutions with the aim of changing the social, physical, economic or legislative environment to make them less conducive to smoking. These are approaches that mainly rely on state or institutional control, either of a link in the supply chain or of smokers' behaviour in the presence of others.

Examples include tobacco crop substitution or diversification, removing subsidies on tobacco production, restricting trade in tobacco products, measures to prevent smuggling, measures to reduce illicit cross-border shopping, restricting advertising of tobacco products, restrictions on selling tobacco products to minors, mandatory health warning labels on tobacco products, increasing the price of tobacco products, restricting access to cigarette vending machines, restricting smoking in the workplace, and restricting smoking in public places. Such approaches could also form part of wider, multifaceted interventions in schools, workplaces or communities.^3^

### Comparator(s)/control

This is a mandatory field

Where relevant, give details of the alternatives against which the main subject/topic of the review will be compared (e.g. another intervention or a non-exposed control group). The preferred format includes details of both inclusion and exclusion criteria.

Control or comparison interventions should be described in as much detail as the intervention being reviewed. If the comparator is ‘treatment as usual’ or ‘standard care’, this should be described, with attention being paid to whether it is ‘standard care’ at the time that an eligible study was done, or at the time the review is done.

Systematic reviews of qualitative studies rarely have a comparator or control; stating ‘Not applicable’ is therefore acceptable.

Examples: Placebo. A group of hospital in-patients who were not exposed to the infectious agent.

### Types of study to be included

This is a mandatory field

Give details of the types of study (study designs) eligible for inclusion in the review. If there are no restrictions on the types of study design eligible for inclusion, or certain study types are excluded, this should be stated. The preferred format includes details of both inclusion and exclusion criteria.

If different study designs are needed for different parts of the review, this should be made clear. Where qualitative evidence will be incorporated in or alongside a review of quantitative data, this should be stated.

Example: We will include randomised trials to assess the beneficial effects of the treatments, and will supplement these with observational studies (including cohort and case–control studies) for the assessment of harms.

### Context

Give summary details of the setting and other relevant characteristics which help define the inclusion or exclusion criteria.

Include relevant details if these form part of the review’s eligibility criteria but are not reported elsewhere in the PROSPERO record.

Examples: Studies in hospital accident and emergency departments. Research in low- and middle-income countries only will be included.

### Main outcome(s)

This is a mandatory field

Give the pre-specified primary (most important) outcomes of the review, including details of how the outcome is defined and measured and when these measurement are made, if these are part of the review inclusion criteria.

For systematic reviews of qualitative studies give details of what the review aims to achieve.

Examples: Change in depression score from baseline to the last available follow-up, measured using the Beck Depression Inventory. Five year progression-free survival (measured from randomisation). Establishing the barriers and facilitators to smoking cessation in pregnancy.

### Additional outcome(s)

This is a mandatory field

List the pre-specified secondary (additional) outcomes of the review, with a similar level of detail to that required for primary outcomes. Where there are no secondary outcomes please state ‘None’ or ‘Not applicable’ as appropriate to the review.

Example: Apgar scores for the baby at 1 and 5 min after birth.

### Data extraction (selection and coding)

This is a mandatory field

Describe how studies will be selected for inclusion. State what data will be extracted or obtained. State how this will be done and recorded.

Data extraction methods reported in systematic review protocols should include:

Study selection.

- The number of reviewers applying eligibility criteria and selecting studies for inclusion in the systematic review (good practice suggests more than one individual) and how this will be done (e.g. whether two people will independently screen records for inclusion or whether one will screen and an other check decisions) and whether researchers will be blinded to each other’s’ decisions.
- How disagreements between individual judgements will be resolved
- The software system or mechanism for recording decisions

Data extraction

- List which data will be extracted from study documents, including information about study design and methodology, participant demographics and baseline characteristics, numbers of events or measures of effect (where applicable). Alternatively, state how this information will obtained from study investigators.
- The number of people extracting or checking received data (good practice suggests more than one individual) and how this will be done (e.g. whether two people will independently extract data or whether one will extract data and an other person check the extracted data).
- How disagreements between individual judgements will be resolved
- How missing data will be handled including whether study investigators will be contacted for unreported data or additional details.
- The means of recording data (e.g. in an excel spreadsheet, in a software system such as Eppi Reviewer)
- Another relevant detail that should be included is the software or tool, if any, that will be used for data extraction and management. An example of such a software tool is the Systematic Review Data Repository-Plus

### Risk of bias (quality) assessment

This is a mandatory field

Describe the method of assessing risk of bias or quality assessment. State which characteristics of the studies will be assessed and any formal risk of bias tools that will be used.

Methods for assessing risk of bias reported in systematic review protocols should include:

- Which characteristics will be assessed (e.g. methods of randomisation, treatment allocation, blinding).
- Whether assessment will be done at study or outcome level
- The criteria used to assess internal validity, if formal a risk of bias assessment is planned (e.g. the Cochrane risk of bias tool, ROBINS, QUADAS).
- How the results of the assessment will inform data synthesis (where applicable).
- The number of reviewers that will be involved in the quality assessment
- How disagreements between reviewers judgements will be resolved

### Strategy for data synthesis

This is a mandatory field

Provide details of the planned synthesis including a rationale for the methods selected. This must not be generic text but should be specific to your review and describe how the proposed analysis will be applied to your data.

Data synthesis methods reported in systematic review protocols should be specific about how they apply to the review and data in question and include:

- Criteria under which the data will be synthesised (e.g. the minimum number of studies or level of consistency required for synthesis)
- Which data will be synthesised including outcomes and summary effect measures (e.g. risk ratios for progression free survival at 2 years)
- The formal method of combining individual study data including, as applicable, information about statistical models that will be fitted (e.g. risk ratios for individual studies will be combined using a random effects meta-analysis) or methods of synthesising qualitative data.

### Analysis of subgroups or subsets

This is a mandatory field

State any planned investigation of ‘subgroups’. Be clear and specific about which type of study or participant will be included in each group or covariate investigated. State the planned analytic approach.

Planned ‘subgroup’ analysis or investigation of potential effect modifiers in reported in systematic review protocols should include:

- The rationale for the investigation (why are differences anticipated, or why is it important to look separately at different types of study or individual)
- Clear definitions of which types of study or individual will be included in each group (e.g. study design such as randomised/ non-randomised trial, intervention type such as high dose/low dose drug, setting such as hospital/ home care, participant characteristics such as male/female, stage III/stage IV tumour, < 18 years/ ≥ 18 years)
- Details of the planned analytic approach (e.g. meta-regression, tests of interaction between groups, logistic regression using individual-level data). Where applicable this should include details of statistical models to be used.

### Type and method of review

This is a mandatory field

Select the type of review and the review method from the lists below. Select the health area(s) of interest for your review.

N.B. The information required here relates to the topic and outcome of the systematic review rather than the methods to be used. It is used to facilitate accurate searching of the database.

### Language

Select each country individually to add it to the list below, use the bin icon to remove any added in error.

The entry will default to English if no other selection is made. For languages other than English, registrants are asked to indicate whether a summary or abstract will be made available in English.

Example: English, French.

### Country

This is a mandatory field

Select the country in which the review is being carried out from the drop down list. For multi-national collaborations select all the countries involved.

Example: England, Canada.

### Other registration details

Give the name of any organisation where the systematic review title or protocol is registered (such as with The Campbell Collaboration, or The Joanna Briggs Institute) together with any unique identification number assigned. (N.B. Registration details for Cochrane protocols will be automatically entered). If extracted data will be stored and made available through a repository such as the Systematic Review Data Repository (SRDR), details and a link should be included here. If none, leave blank.

Example: The title for this review and the review protocol are recorded in the Campbell Library as Project 27.

### Reference and/or URL for published protocol

Give the citation and link for the published protocol, if there is one. This may be to an external site such as a journal or organisational website. Alternatively an unpublished protocol may be deposited with CRD in pdf format. A link to this will be automatically added.

Example: Free C, Phillips G, Felix L, Galli L, Patel V, Edwards P. The effectiveness of M-health technologies for improving health and health services: a systematic review protocol. BMC Research Notes 2010, 3:250 doi:10.1186/1756-0500-3-250http://www.biomedcentral.com/1756-0500/3/250.

### Dissemination plans

Give brief details of plans for communicating essential messages from the review to the appropriate audiences. Any knowledge transfer or implementation activities beyond publication of the final report that are planned should be included.

Example: In addition to producing a report for the funders of this review, which will be made available free of charge on their website, a paper will be submitted to a leading journal in this field. Furthermore, should the findings of the review warrant a change in practice, a one page summary report will be prepared and sent to lead clinicians and healthcare professionals in the National Health Service.

### Keywords

Give words or phrases that best describe the review. Keywords will help users find the review in the Register (the words do not appear in the public record but are included in searches). Be as specific and precise as possible. Avoid acronyms and abbreviations unless these are in wide use.

The addition of keywords is particularly important for non-effectiveness reviews. These records are likely to contain fewer relevant terms in other fields such as comparators and outcomes.

Information specialists at the Centre for Reviews and Dissemination (CRD) will assign MeSH terms, which will appear in the public record.

Example: Systematic review; meta-analysis; recurrence; survival; radiation; resectable; soft-tissue; sarcoma.

### Details of any existing review of the same topic by the same authors

Give details of earlier versions of the systematic review if an update of an existing review is being registered, including full bibliographic reference if possible.

Example: This review is an update of our earlier systematic review and economic model and is being undertaken in the light of the publication of significant new research which will assist in developing our model. The citation for the existing review is Fayter D, Nixon J, Hartley S, Rithalia A, Butler G, Rudolf M, Glasziou P, Bland M, Stirk L, Westwood M. A systematic review of the routine monitoring of growth in children of primary school age to identify growth-related conditions. *Health Technol Assess*. 2007;11(22):1–87.

### Current review status

This is a mandatory field

Review status should be updated when the review is completed and when it is published.

Select from the list below to indicate the current status of the review.

Use the free text box to provide an explanation of the status of the review.

Example: Discontinued: This review has been abandoned as we have been unable to secure adequate funding to proceed.

### Any additional information

Provide any other information the review team feel is relevant to the registration of the review.

Example: This review is being undertaken as part of the planning for a randomised trial to compare all different types of radiotherapy for localised, resectable soft-tissue sarcoma.

### Details of final report/publication(s) or preprints if available

This field should be left empty until details of the completed review are available OR you have a link to a preprint.

Give the full citation for the preprint or final report or publication of the systematic review, including the URL where available.

This field may also be used to record the availability of an un-published final report, summary results etc.

Example: Toulis KA, Goulis DG, Venetis CA, Kolibianakis EM, Negro R, Tarlatzis BC, Papadimas I. Risk of spontaneous miscarriage in euthyroid women with thyroid autoimmunity undergoing IVF: a meta-analysis. Eur J Endocrinol. 2010 Apr;162(4):643- 52. Epub 2009 Dec 2. http://eje-online.org/cgi/content/full/162/4/643
